# Adjuvant therapeutic effects and mechanisms of Yinchenhao decoction in obstructive jaundice: a narrative review

**DOI:** 10.3389/fphar.2026.1811123

**Published:** 2026-05-21

**Authors:** Si-Han Che, Saud Ahmad Saad, Mao-Lin Ju, Zhen You

**Affiliations:** 1 Division of Biliary Surgery, Department of General Surgery, West China Hospital, Sichuan University, Chengdu, Sichuan, China; 2 Research Center for Biliary Diseases, West China Hospital, Sichuan University, Chengdu, Sichuan, China; 3 Affiliated Hospital of Southwest Medical University, Southwest Medical University, Luzhou, Sichuan, China

**Keywords:** bile acid metabolism, herbal formula, liver fibrosis, obstructive jaundice, Yinchenhao decoction

## Abstract

Yinchenhao Decoction (YCHD) is a classic traditional Chinese medicine (TCM) formula composed of three botanical drugs: *Artemisia capillaris* Thunb, *Gardenia jasminoides* Ellis, and *Rheum palmatum* L. In modern research, it has demonstrated promising therapeutic effects against various diseases, including obstructive jaundice (OJ), hepatitis, liver cirrhosis, and hepatic carcinoma. From the perspective of TCM theory, YCHD possesses the properties of clearing heat and detoxifying as well as promoting diuresis to resolve jaundice, and is commonly used for the treatment of “damp-heat type” jaundice, particularly OJ. This article reviews the efficacy, main active metabolites, and pharmacokinetic characteristics of YCHD as an adjuvant therapy for OJ, and analyzes in depth its multi-target mechanism of action, such as regulating bile acid (BA) metabolism, reducing liver cell damage, exerting anti-inflammatory effects, and regulating metabolic processes. Furthermore, it proposes reference suggestions for improving the practical application of YCHD from both basic research and clinical research perspectives.

## Graphical Abstract

**Figure:**
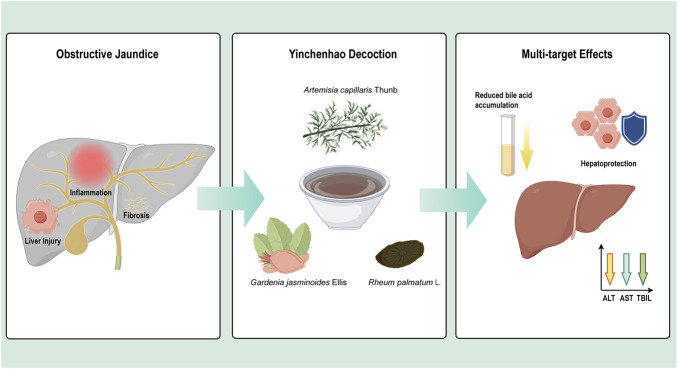

## 1 Introduction

Jaundice is a condition characterized by elevated serum bilirubin levels, resulting in yellowing of the sclera, skin, and mucous membranes. Obstructive jaundice (OJ) is caused by impaired bile outflow due to intrahepatic or extrahepatic factors, with bile duct stones and biliary tract malignant tumors being the primary etiologies. The intrahepatic bile stasis caused by OJ can cause a lot of damage to the liver. OJ can lead to complications such as cholangitis, bile reflux, and bacteremia (Pavlidis and Pavlidis, 2018; Rizzo et al., 2020; Zhang et al., 2022). In severe cases, it may progress to liver cirrhosis and renal failure (Jorge et al., 2016; Alabdul et al., 2025). Currently, invasive surgical treatment remains the mainstay for OJ (Chandrashekhara et al., 2016; Kang et al., 2024). Ursodeoxycholic acid (UDCA) and Obeticolic acid (OCA) are among the few postoperative treatment drugs for OJ, yet these therapeutic approaches have notable limitations. For instance, some patients are non-responsive to UDCA, and OCA use has been associated with a higher risk of complications compared to placebo (Suraweera et al., 2017; Feldman and Sokol, 2019). Yinchenhao Decoction (YCHD), recorded in the Treatise on Febrile Diseases (Shang Han Lun), a classic TCM text written by Zhang Zhongjing, is officially included in the Chinese Pharmacopoeia. For over 1,800 years, it has been an indispensable classic formula for treating OJ and liver diseases (Wang et al., 2013). From the TCM perspective, YCHD possesses the properties of clearing heat and detoxifying, as well as promoting diuresis to resolve jaundice. Cholestatic symptoms observed in diseases such as hepatic fibrosis, liver cirrhosis, and hepatitis are mostly categorized as “Damp-heat style (Shi Re Zheng)” jaundice (Cai et al., 2020), and the pathological characteristics of OJ align perfectly with the therapeutic indications of YCHD. According to modern pharmacological studies (Cai et al., 2020; Li et al., 2017), YCHD regulates bile acid (BA) and substance metabolism, inhibits inflammation, and exerts hepatoprotective effects, which can effectively inhibit or even reverse the series of damages caused by OJ. Therefore, although the evidence base needs to be rigorously evaluated, YCHD as an adjuvant therapy after OJ surgery may have certain advantages. Although several studies have explored the mechanisms and efficacy of YCHD in treating OJ, a comprehensive review on this topic is lacking. This study summarizes the latest research progress of YCHD in assisting OJ treatment, focusing on three aspects: qualitative and quantitative analysis of its active metabolites, molecular mechanisms of its therapeutic effect on OJ, and clinical evidence supporting its application, in order to provide new directions for postoperative drug treatment and research of OJ. To ensure a comprehensive overview, We performed a systematic search in PubMed and Web of Science databases using the keywords ‘Yinchenhao decoction’, ‘obstructive jaundice’, and ‘cholestasis’ from inception to January 2026. The search was limited to English-language original research articles and peer-reviewed reviews. Additional studies were identified by manual screening of reference lists of included articles and relevant reviews. The final selection was based on relevance to the therapeutic mechanisms, active metabolites, or clinical application of YCHD in obstructive jaundice.

## 2 Composition and active molecules of YCHD

In TCM theory, based on the differences in medicinal importance and functions, a formula principle known as “Jun-Chen-Zuo-Shi (monarch, minister, assistant, and guide)” has been established to characterize the roles of different metabolites in a prescription. In modern scientific research, methods such as chemical component identification and pharmacokinetic detection are commonly employed to screen for major active substances. The “Jun-Chen-Zuo-Shi” formulation principle was first recorded in the Huangdi Neijing (Yellow Emperor’s Internal Classic). Drugs in a formula can be classified into four categories according to their functions: monarch drug, minister drug, assistant drug, and guide drug, collectively referred to as Jun, Chen, Zuo, and Shi. The monarch drug plays the primary therapeutic role in the formula, the minister drug assists the monarch drug in exerting its therapeutic effects, and the assistant drug functions to reduce toxicity and enhance efficacy (Li et al., 2017). YCHD is composed of *Artemisia capillaris* Thunb (Yinchen), *Gardenia jasminoides* Ellis (Zhizi), and *Rheum palmatum* L. (Dahuang). Yinchen serves as the monarch drug of this formula. According to TCM theory, it possesses the effects of clearing heat, eliminating dampness, protecting liver function, and alleviating jaundice (Wang et al., 2021). Zhizi acts as the minister drug, which is widely used in TCM for its functions of clearing heat, relieving vexation, cooling blood, and promoting blood circulation to remove stasis (Chen et al., 2020). Dahuang functions as both the assistant and guide drug, honored as the “general” in TCM botanical drugs. Its main effects include protecting the liver, inhibiting oxidation, fibrosis, and cirrhosis, and it is used to treat liver failure, hepatocellular carcinoma, and various types of hepatitis (Li et al., 2017; Zhuang et al., 2020).

While TCM uses the “Jun-Chen-Zuo-Shi” principle to understand the roles of different metabolites in a formula, this alone is insufficient for an in-depth exploration of drug mechanisms. Investigating the chemical composition and major active substances of drugs is the first step in in-depth research and also the basis for formulating treatment plans in modern medicine. All three constituent botanical drugs of YCHD contain complex chemical metabolites (Li et al., 2017). The main types of active substances in Yinchen include coumarins, flavonoids, chromones, organic acids, and alkaloids (Cai et al., 2020). The major active component types in Zhizi are gardenosides, pigments, organic acids, volatile oils, and gardenia polysaccharides (Chen et al., 2020). The main active metabolites of Dahuang fall into five categories: anthraquinones, tannins, stilbenes, volatile oils, and rhubarb polysaccharides (Zhuang et al., 2020). Notably, scoparone (6,7-dimethoxycoumarin), a key active component in the monarch drug Yinchen, exhibits choleretic, anti-inflammatory, and hepatoprotective activities, and has received increasing attention for its therapeutic efficacy in liver diseases. Studies have found that the combination of YCHD exerts a more potent therapeutic effect than any single component or combination of two metabolites. Therefore, the interactions and synergistic effects among the drug metabolites may contribute significantly to the therapeutic efficacy of YCHD. Some studies have suggested that further research on the dose-response relationship of scoparone is warranted (Wang et al., 2013; Hui et al., 2020). The specific chemical metabolites are presented in Table 1.

**TABLE 1:** Potentially active metabolites of the botanical drugs in YCHD.

Herb (Latin name)	Common name	Primary active components	Representative compounds (Examples)	Pharmacological relevance	Key molecular targets/pathways	Ref.
*Artemisia capillaris* Thunb.	Yinchen	Coumarins	Scoparone, 6,7-Dimethylesculetin	Choleretic, hepatoprotective, anti-inflammatory, antioxidant	CAR, Nrf2, PPARγ, NF-κB, CYP3A, UGT1A1, SULT2A1, MRP2/3/4	Cai et al. (2020)
		Flavonoids	Arcapillin, Cirsimaritin, 3'-Methoxy thistle flavin	Antioxidant, anti-inflammatory	Nrf2, NF-κB	
		Chromones	Capillarisin, 7-Methylcapillarisin, 4'-Methylcapillarisin	Choleretic, hepatoprotective	CAR, MRP2	
		Organic acids	Chlorogenic acid, Coumaric acid A and B	Antioxidant	Nrf2	
		Alkaloids, Terpenes, Peptides	Capillin, Monoterpenes, Sesquiterpenes, Water-soluble polypeptides	Anti-inflammatory, immunomodulatory	NF-κB, cytokines	
*Gardenia jasminoides* Ellis	Zhizi	Iridoid glycosides	Geniposide, Hydroxygeniposide, Geniposidic acid, Gardoside, Scandoside methyl ester	Anti-inflammatory, regulation of bile acid transporters	FXR, BSEP, MRP2, OATP1A4, UGT1A1	Chen et al. (2020)
		Pigments	Crocin, Crocetin	Antioxidant, hepatoprotective	Nrf2, HO-1	
		Organic acids	Chlorogenic acid, Crocetinic acid	Anti-inflammatory	NF-κB	
		Volatile oils	Linoleic acid, Palmitic acid	/	​	
		Polysaccharides	Gardenia polysaccharides	Immunomodulatory	Macrophage regulation	
*Rheum palmatum* L.	Dahuang	Anthraquinones	Rhein, Emodin, Aloe-emodin, Chrysophanol, Physcion,Sennosides	Anti-fibrotic, anti-apoptotic, purgative, anti-inflammatory	PI3K/Akt, PERK-CHOP-GADD34, TGF-β1, α-SMA, Bax/Bcl-2, caspase-3	Zhuang et al. (2020)
		Tannins	Gallic acid, D-Catechin	Antioxidant, hepatoprotective	Nrf2	
		Stilbenes	Rhaponticin, Piceatannol, Resveratrol	Anti-inflammatory, anti-fibrotic	SIRT1, NF-κB	
		Volatile oils	Palmitic acid, Ethyl palmitate	/	​	
		Polysaccharides	Rhubarb polysaccharides	Immunomodulatory	Macrophage, gut microbiota	

Considering pharmaceutical processes and drug metabolism, only a portion of these metabolites may exert therapeutic effects, and they may undergo changes during these processes. Additionally, TCM formulas are characterized by multi-component and multi-target actions, making stability and quality control particularly crucial. The determination of active metabolites in TCM is a primary method for quality control (Jiang et al., 2020a). Therefore, analyzing the active substances that exert effects from serological and pharmacokinetic perspectives is more scientific.

To improve the reproducibility of YCHD-related studies, quantitative information on its major active metabolites is essential. Yi et al. performed a simultaneous quantification of 14 active metabolites in YCHD using UHPLC-DAD (Yi et al., 2018). In their study, YCHD was prepared according to the classical ratio (Yinchen:Zhizi:Dahuang = 18:12:6, w/w/w), decocted twice, and concentrated to a final concentration of 1 g crude drug/mL. The five most abundant metabolites were geniposide (9,274 μg/mL), genipin-1-β-D-gentiobioside (2,242 μg/mL), caffeic acid (631.7 μg/mL), chlorogenic acid (280.1 μg/mL), and rhein (260.0 μg/mL). These quantitative data can serve as quality control benchmarks for future studies, as the content of active metabolites may vary depending on the source of raw botanical drugs and preparation conditions.

### 2.1 *In vitro* and *in vivo* determination of component types in YCHD

Previously, we separately introduced the main active metabolites of the three constituent botanical drugs of YCHD. However, the key substances through which YCHD exerts its effects in the human body are by no means a simple sum of the metabolites of these three botanical drugs, as these active substances undergo numerous changes during pharmaceutical processing and absorption. Studies have confirmed that the active metabolites entering the circulatory system or target tissues/organs, as well as metabolites derived from these active metabolites, are typically the effective therapeutic substances of traditional Chinese medicines (TCMs) (Liu S. et al., 2022). Therefore, research on the effective metabolites of TCMs must focus on metabolites in organisms. In recent years, when investigating the effective active substances of YCHD, researchers often use serum or tissue extracts from relevant animals, with mass spectrometry as the core analytical technology.

Using Ultra Performance Liquid Chromatography/Quadrupole Time-of-Flight Mass Spectrometry (UPLC/Q-TOF/MS) to chemically profile the YCHD formula, Wang et al. (Wang et al., 2008) successfully identified 30 metabolites. The monarch drug Yinchen mainly contributed coumarins and flavonoids, the minister drug Zhizi primarily contained iridoid glycosides, and the assistant-guide drug Dahuang was rich in anthraquinones. Furthermore, they identified 21 compounds derived from YCHD in the plasma of rats orally administered YCHD, including 19 parent compounds directly absorbed into the bloodstream and 2 metabolites. This study explored several compounds of YCHD *in vivo* but did not analyze the distribution of prototype and derived metabolites of YCHD in target tissues and organs. To address this limitation, subsequent studies improved the detection methods and further analyzed samples such as serum and tissues from rats orally administered YCHD (Wang et al., 2024). In this study, a total of 139 compounds were detected in the *in vitro* analysis of YCHD, among which 36 were further confirmed using reference standards. The researchers then conducted comprehensive detection of plasma, excreta, bile, and organ tissues (spleen, liver, brain, lung, intestine, kidney, heart) from YCHD-treated rats, identifying 58 parent compounds and 175 metabolites, which together constitute the *in vivo* metabolic correlation group of YCHD. Both phase I and phase II metabolic reactions were extensively involved. In the liver-the primary target organ where YCHD exerts hepatoprotective and choleretic effects-12 parent compounds and 11 metabolites were detected, including iridoid glycosides, anthraquinones, and organic acids, with key metabolites such as geniposide, rhein, and emodin identified. In bile, 4 parent compounds and 12 metabolites were found, indicating that compounds processed by the liver and their metabolites can be excreted into bile and participate in the “enterohepatic circulation”. In the intestine, 5 parent compounds and 8 metabolites were detected; notably, the presence of genipin in the intestine is crucial for regulating intestinal barrier function and flora balance. In feces, 26 parent compounds and 28 metabolites were identified, many of which are unabsorbed parent compounds or products metabolized by intestinal flora, suggesting that intestinal flora metabolism is an important link in the efficacy of YCHD. Urine serves as the primary route for metabolite excretion, with a large number of metabolites detected, indicating that the kidney plays a major role in clearing highly water-soluble phase II conjugates (Wang et al., 2024).

To date, research on the active metabolites of YCHD has progressed through three stages: *in vitro* determination, serological detection, and target tissue/organ-level analysis, leading to a relatively in-depth understanding of its effective metabolites and quality control markers. However, as the physiological and pathological states of organisms change, the types and distribution characteristics of drug active metabolites may also vary accordingly. Future studies should fully consider this factor when constructing experimental animal models.

### 2.2 Pharmacokinetic studies of YCHD

**FIGURE 1:**
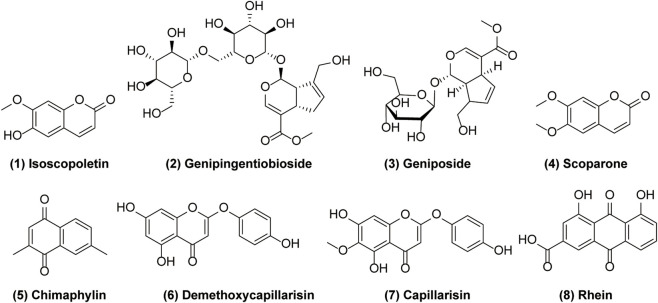
Chemical structures of candidate active metabolites in YCHD. 1. Isoscopoletin; 2. Genipingentiobioside; 3. Geniposide; 4. Scoparone; 5. Chimaphylin; 6. 6-demethoxycapillarisin; 7. Capillarisin; 8. Rhein.

Only compounds absorbed into the bloodstream can exert biological activities, but therapeutic effects require maintaining a certain concentration for a sufficient duration. Therefore, pharmacokinetic research is of great value for exploring the actual substances responsible for the therapeutic efficacy of YCHD. Wang et al. (2011) developed an Ultra Performance Liquid Chromatography-Electrospray Ionization-Quadrupole Time-of-Flight Tandem Mass Spectrometry (UPLC-ESI-Q-TOF-MS/MS) method to analyze the pharmacokinetic parameters of 21 active substances of YCHD in serum. By comprehensively evaluating pharmacokinetic parameters (AUC, C_max_, t_1/2_, etc.) and combining TCM compatibility theory, 9 candidate active metabolites were initially screened, including: 1 (7-methoxycoumarin-6-hydroxyl), 2 (genipin gentiobioside), 3 (geniposide), 4 (6,7-dimethylesculetin), No.16 (Not identified), 5 (chimaphylin), 6 (6-demethoxycapillarisin), 7 (capillarisin), and 8 (rhein). Notably, 7-Methoxycoumarin-6-hydroxyl is a metabolite of Scoparone. These substances may be the key active metabolites or their metabolites responsible for the main effects of YCHD, and their chemical structures are presented in Figure 1. The pharmacokinetic parameters of the corresponding active ingredients are shown in Table 2.

**TABLE 2:** Partial pharmacokinetic parameters of YCHD core active metabolites in rats after oral administration (10 g/kg, n = 10).

Compound	Tmax (h)	Cmax (mg/L)	AUC (mg·h/L)	T1/2/h
Isoscopoletin	1.52	0.34	1.36	10.04
Genipin gentiobioside	0.12	1.43	1.08	4.75
Geniposide	0.35	20.04	2765.1	1.13
Scoparone	5.13	30.94	11169.89	0.96
Chimaphylin	5.28	2.09	190.72	15.67
Demethoxycapillarisin	1.12	0.25	10.38	1.6
Capillarisin	0.65	0.49	1.92	0.44
Rhein	1.92	0.07	1.09	23.84

T_max_, Time to peak drug concentration; C_max_, Peak concentrations; AUC, Area under the concentration-time curve; T_1/2_, Half-life period.

Studying the pharmacokinetics of drugs is an important reference for formulating administration regimens. Due to the multi-component nature of TCMs, different active substances exhibit distinct metabolic characteristics. In recent years, the strategy of “integrated pharmacokinetics of multi-component TCMs” has been adopted by some researchers to address this issue. This approach determines the pharmacokinetic weight coefficient of each component by calculating the Area Under the Concentration-Time Curve (AUC), then uses mathematical models to integrate multiple metabolites to obtain an integrated plasma concentration-time curve that characterizes the overall behavior of the TCM. Ultimately, comprehensive pharmacokinetic parameters are calculated to develop an administration regimen suitable for the corresponding preparation (Shi et al., 2018; Lu et al., 2021; Ma et al., 2024). Conducting integrated pharmacokinetic research on YCHD may represent a direction for further optimizing its administration regimen. In addition, pharmacokinetic analysis based solely on serology is insufficient. After being absorbed into the bloodstream, drugs exhibit differential distribution across various organs and tissues, with variations in both component types and concentrations. A previous study (Wang et al., 2024) has conducted qualitative detection of YCHD metabolites in plasma, excreta, bile, and organ tissues (spleen, liver, brain, lung, intestine, kidney, heart), but corresponding quantitative detection remains lacking. Quantitative detection of YCHD active substances based on different distribution scenarios will bring greater value to the investigation of YCHD’s mechanism of action and its clinical application.

## 3 Mechanisms of YCHD in the treatment of obstructive jaundice

OJ triggers a cascade of pathological events beginning with bile acid (BA) accumulation in the liver (Molinaro and Marschall, 2022), which subsequently induces inflammatory and oxidative stress responses, and eventually leads to hepatocyte apoptosis and hepatic fibrosis (Jorge et al., 2016; Perez and Briz, 2009). Beyond hepatic impairment, OJ has widespread systemic effects. Without timely intervention, OJ can cause portosystemic shunting and systemic BA toxicity, resulting in systemic inflammatory response, renal injury, and even multiple organ failure (Pavlidis and Pavlidis, 2018; Liu et al., 2021; Liu et al., 2023). YCHD exerts multi-target therapeutic effects that intervene at each of these progressive stages. Based on this pathological framework, the mechanisms of YCHD against OJ are summarized into three interconnected aspects: (1) regulating BA synthesis, metabolism, and excretion; (2) inhibiting inflammatory and oxidative damage; and (3) suppressing hepatocyte apoptosis and hepatic fibrosis.

### 3.1 Regulating the synthesis, metabolism, and excretion of bile acids (targeting cholestasis)

**FIGURE 2:**
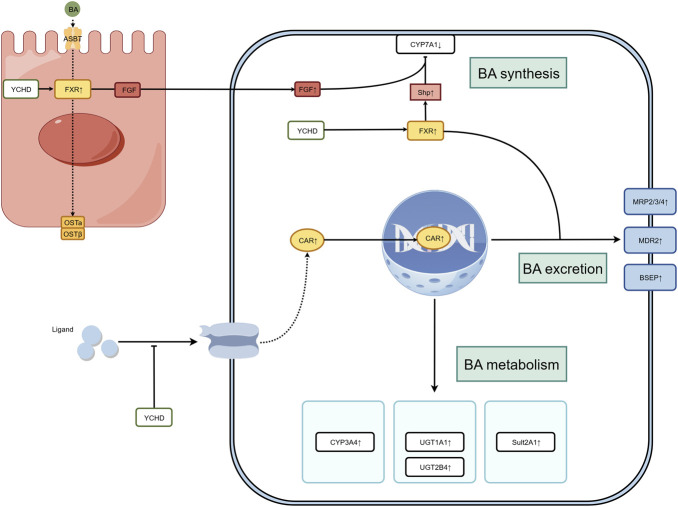
Mechanisms underlying YCHD-mediated alleviation of OJ by regulating bile acid metabolism.

YCHD alleviates cholestasis—the initial trigger of OJ pathology—by modulating BA homeostasis through multiple signaling pathways and transporters. The relevant mechanisms are illustrated in Figure 2.

EGFR-CAR axis: Liu et al. (2025) demonstrated that YCHD mitigates OJ-induced liver injury by inhibiting epidermal growth factor receptor (EGFR) activation, promoting constitutive androstane receptor (CAR) nuclear translocation, and subsequently upregulating BA-metabolizing enzymes, including cytochrome P450 3A (CYP3A), UDP-glucuronosyltransferase 1-1 (UGT1A1), and sulfotransferase 2A1 (SULT2A1), as well as efflux transporters such as multidrug resistance-associated protein 2 (MRP2), MRP3, and MRP4.

Regulation of BA transporters and enzymes: Yi et al. (2018) showed that in ANIT-induced cholestatic rats, YCHD restores the expression of UGT1A1, MRP2, bile salt export pump (BSEP), and organic anion-transporting polypeptide 1A4 (OATP1A4), thereby promoting bilirubin and BA excretion.

FXR-FGF15 axis (gut-liver crosstalk): Li et al. (2025a) found that YCHD remodels intestinal flora to enrich secondary BAs (CDCA, DCA, CA), which activate the intestinal farnesoid X receptor (FXR)-fibroblast growth factor 15 (FGF15) axis, leading to inhibition of hepatic cholesterol 7α-hydroxylase (Cyp7a1) expression and reduced BA synthesis. YCHD also preserves intestinal barrier integrity, limiting bacterial and toxin translocation.

BAs are synthesized from cholesterol in the liver and are essential for cholesterol catabolism and nutrient absorption (Russell, 2003). Their homeostasis is maintained by a network of transporters—such as MRP2, MRP3, MRP4, and BSEP—which mediate BA excretion into bile or reflux into circulation (Keppler, 2014; Muchova et al., 2015; Alaei Faradonbeh et al., 2022; Susukida et al., 2015). Dysregulation of BA homeostasis is associated with various enterohepatic and metabolic diseases, including cholestasis, non-alcoholic steatohepatitis, inflammatory bowel disease, and obesity (Xiang et al., 2023). The FXR serves as a master regulator of BA homeostasis by transcriptionally controlling genes involved in BA synthesis, transport, and enterohepatic circulation (Xiang et al., 2023; Sun L. et al., 2021; Radun and Trauner, 2021). Additionally, the intestinal microbiota modulates BA composition by producing secondary BAs that activate FXR and support intestinal barrier function (Ramírez-Pérez et al., 2017). Specific intestinal flora can enrich secondary BAs (CDCA, DCA, CA) that activate FXR and protect the integrity of the intestinal barrier (Li et al., 2025a), exerting a positive regulatory effect on BA metabolism.

### 3.2 Inhibiting inflammatory oxidative damage (targeting inflammation and oxidative stress)

**FIGURE 3:**
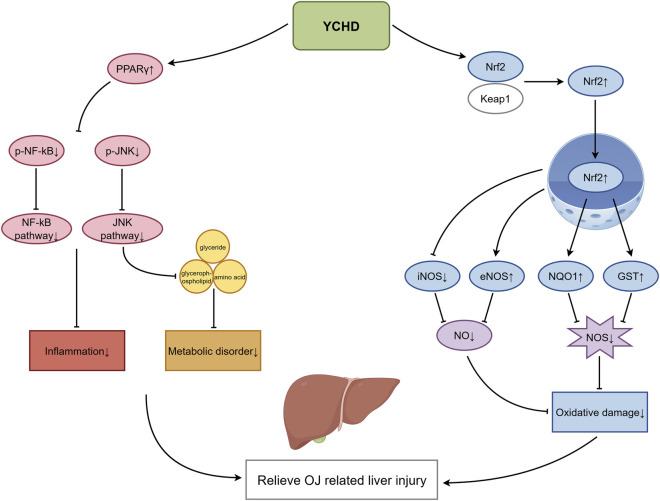
Mechanisms of YCHD in inhibiting inflammatory oxidative damage and modulating metabolic disorders.

Following BA accumulation, inflammatory and oxidative stress responses exacerbate liver injury. The relevant mechanisms are illustrated in Figure 3. YCHD counteracts these processes through the following pathways:

PPARγ/NF-κB/JNK pathway (anti-inflammatory): Li et al. (2025b) showed that YCHD metabolites (e.g., emodin, geniposide, quercetin) regulate the PPARγ/NF-κB/JNK pathway, suppress inflammation, and restore glycerolipid, glycerophospholipid, and amino acid metabolism. This pathway also restores disturbed glycerolipid, glycerophospholipid, and amino acid metabolism.

Nrf2-Keap1 pathway (antioxidant): Liu J-J. et al. (2022) further demonstrated that YCHD activates the nuclear factor erythroid 2-related factor 2 (Nrf2) pathway, promoting nuclear translocation of Nrf2 and upregulating antioxidant enzymes such as NAD(P)H quinone dehydrogenase 1 (NQO1) and glutathione S-transferase (GST). This enhances ROS scavenging and modulates nitric oxide metabolism by reducing inducible nitric oxide synthase (iNOS) and increasing endothelial nitric oxide synthase (eNOS) expression, thereby limiting oxidative injury.

OJ causes the abnormal accumulation of bile metabolites in the liver and systemic circulation, leading to lipid metabolism disorders and inflammatory oxidative damage in the body (Gao et al., 2024; Li et al., 2022). These two pathological changes have been confirmed to be closely associated with the development and progression of diseases such as fatty liver, enterohepatic inflammation, atherosclerosis, and hepatic fibrosis (Aranda et al., 2013; Rana et al., 2019; Bao et al., 2025). Among these, The Nrf2-Keap1 pathway, a central antioxidant defense system, is often impaired in cholestasis. By restoring this pathway and reducing inflammation, YCHD helps prevent progression to more severe conditions such as steatosis and fibrosis.

### 3.3 Inhibiting hepatocellular apoptosis and hepatic fibrosis (targeting end-stage liver damage)

**FIGURE 4:**
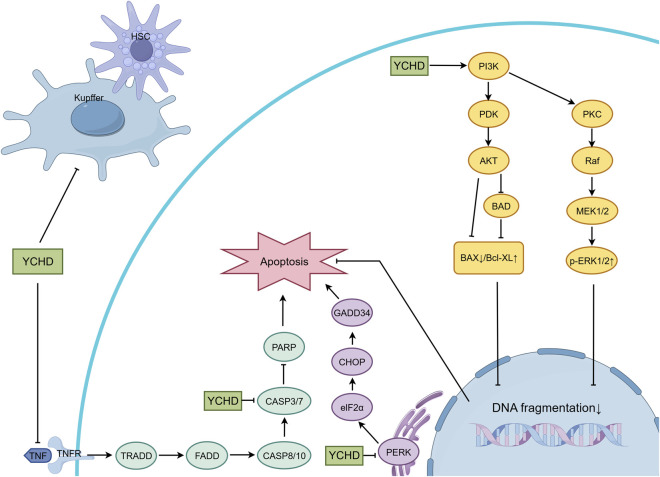
Mechanisms of YCHD in inhibiting hepatocellular apoptosis and hepatic fibrosis.

In the middle and late stages of OJ, persistent injury leads to hepatocyte apoptosis and activation of hepatic stellate cells (HSCs), resulting in fibrosis. The relevant mechanisms are illustrated in Figure 4. YCHD intervenes through the following mechanisms.

#### 3.3.1 Fibrosis pathways

HSC activation: Liu et al. (2008) reported that YCHD reduces HSC activation, evidenced by decreased alpha-smooth muscle actin (α-SMA) expression.

Kupffer cell-mediated inflammation: YCHD downregulates CD68 (a Kupffer cell marker) and profibrotic factors (TGF-β1, TNF-α), modulating the inflammatory microenvironment that drives fibrosis.

#### 3.3.2 Apoptosis pathways

PI3K-Akt pathway: Cai et al. (2019) identified that YCHD metabolites such as emodin and quercetin suppress TNF-α-induced apoptosis in hepatocytes by modulating the PI3K-Akt pathway, reducing cleaved caspase-3, and increasing p-ERK1/2, PI3K, and Bcl-XL.

PERK-CHOP-GADD34 pathway: Wu et al. (2019) further demonstrated that YCHD alleviates OJ-induced apoptosis by inhibiting the PERK-CHOP-GADD34 pathway and restoring the Bax/Bcl-2 balance.

Bax/Bcl-2 balance: YCHD restores the anti-apoptotic/pro-apoptotic balance, reducing cytochrome c release and caspase-3 activation.

Hepatic stellate cells (HSCs) and Kupffer cells play critical roles in hepatic fibrosis. Regulating these cells is essential for blocking hepatic fibrosis and reversing the progression of liver cirrhosis (Gao et al., 2022; Horn and Tacke, 2024). Apoptosis can be triggered through two main pathways: the extrinsic pathway is activated by Fas receptors and directly initiates apoptosis via caspase-8; the intrinsic pathway is mediated by mitochondria. When the pro-apoptotic factor Bax increases membrane permeability, cytochrome C is released, which in turn activates downstream caspases. In cholestasis, BAs can not only activate Fas receptors but also induce apoptosis by damaging mitochondria and disrupting the balance between Bax and the anti-apoptotic factor Bcl-2 (Soltani et al., 2023; Ahsan et al., 2024). Activated caspase-3 not only directly induces cell death but also cleaves the N-terminal domain of gasdermin E (GSDME), disrupts cell membranes, and releases inflammatory factors, thereby mediating pyroptosis (Jiang et al., 2020b; Bhat et al., 2023). Activation of the PI3K/AKT pathway exerts a protective effect against Fas- or TNF-α-induced hepatocellular death and liver injury (Liu et al., 2019). Additionally, the PERK-CHOP-GADD34 pathway also plays an important role in apoptosis (Wu et al., 2023).

### 3.4 Overview of integrated mechanisms and synergistic effects

In summary, YCHD exerts synergistic therapeutic effects against the complex pathological network of OJ through its multi-component properties. Its core active metabolites (e.g., coumarins from Yinchen, iridoid glycosides from Zhizi, anthraquinones from Dahuang) act on three core pathological processes—cholestasis, inflammatory oxidative damage, and apoptosis/fibrosis—by regulating multiple key targets and pathways, ultimately leading to improved liver function and reduced hepatic injury (Table 3).

**TABLE 3:** Integrated mechanisms of Yinchenhao decoction: active metabolites, targets, and therapeutic outcomes in obstructive jaundice.

Pathological process	Key active components (Examples)	Targets/Pathways involved	Ref.
Bile acid metabolic disorder	Geniposide, Rhein, Scoparone, etc	1. Nuclear receptors and enzymes/transporters: activates CAR, upregulates CYP3A, UGT1A1, SULT2A1, MRP2/3/4, BSEP, OATP1A4.	Yi et al. (2018), Liu et al. (2025), Li et al. (2025a)
		2. Gut-liver axis: modulates gut microbiota, activates intestinal FXR-FGF15 axis, inhibits hepatic Cyp7a1.	
Inflammation and oxidative damage	Emodin, Geniposide, Quercetin, etc.	1. Inflammatory pathways: upregulates PPARγ, inhibits phosphorylation of NF-κB and JNK.	Li et al. (2025b), Liu J-J. et al. (2022)
		2. Antioxidant pathways: activates Nrf2, upregulates NQO1, GST; balances iNOS/eNOS.	
Hepatocyte apoptosis and fibrosis	Emodin, Kaempferol, Quercetin, etc.	1. Apoptosis pathways: inhibits PERK-CHOP-GADD34 pathway; modulates Bax/Bcl-2 balance; inhibits caspase-3 activation.	Liu et al. (2008), Cai et al. (2019), Wu et al. (2019), Gao et al. (2022)
		2. Fibrosis pathways: inhibits HSC activation (↓α-SMA); suppresses Kupffer cell function (↓CD68) and expression of TGF-β1, TNF-α.	

## 4 Preclinical and clinical evidence

YCHD has been used for over 1,800 years to treat damp-heat type jaundice. With the advent of modern surgical and pharmacological treatments for OJ, YCHD continues to serve as an important complementary therapy. It improves clinical symptoms and liver function, reduces hepatic inflammation and fibrosis, enhances patients’ quality of life, and is characterized by high safety and low toxicity (Wu et al., 2015). In recent years, a substantial body of preclinical studies (including both *in vitro* and *in vivo* models) and clinical evidence has further validated the therapeutic effects of YCHD on OJ.

### 4.1 Preclinical evidence: *in vivo* and *in vitro* studies

Preclinical investigations have employed various animal models and cell-based systems to elucidate the efficacy and mechanisms of YCHD against OJ.

#### 4.1.1 *In vivo* animal models

The most commonly used animal models for OJ and cholestasis include bile duct ligation (BDL) in rats and chemical-induced cholestasis using alpha-naphthylisothiocyanate (ANIT) in rodents. These models reliably reproduce key features of OJ, including elevated serum bilirubin and bile acids, hepatocellular necrosis, inflammation, and subsequent fibrosis. Table 4 summarizes representative preclinical studies.

**TABLE 4:** Key preclinical studies of YCHD in obstructive jaundice/cholestasis: intervention type, dosage, and outcomes.

Study	Model	Experimental group	Control group	Dosage/Concentration	Key outcomes
Yi et al. (2018)	ANIT-induced rat	Complete YCHD decoction	Normal saline	12 g/kg/d (oral, high dose)	↑ UGT1A1, MRP2, BSEP, OATP1A4; ↓ TBIL, DBIL, ALT, AST; ↑ bile flow rate; improved histopathology
Liu et al. (2025)	BDL rat	Complete YCHD decoction	Normal saline	3.6 g/kg/d (oral; 1.8 mL/kg bid, 1 g/mL)	↓ liver injury; ↑ CAR nuclear translocation; ↑ CYP3A4, UGT1A1, MRP2/3/4 (liver), MRP2/4 (kidney); improved bile acid clearance
Li et al. (2025a)	ANIT-induced rat	Complete YCHD decoction	Normal saline	3 or 9 g/kg/d (oral)	Remodeled gut microbiota (↑ Roseburia, Parasutterella; ↓ Escherichia-Shigella); ↑ secondary BAs (CDCA, DCA, CA); activated intestinal FXR-FGF15; ↓ hepatic Cyp7a1; restored intestinal barrier (Occludin, Claudin-1)
Liu et al. (2022a)	BDL rat	Complete YCHD decoction	Normal saline	3.6 g/kg/d (oral; 3.6 mL/kg, 1 g/mL)	↓ TBIL, DBIL, ALT, AST; activated Nrf2 nuclear translocation; ↑ NQO1, GST; ↓ iNOS, ↑ eNOS; reduced oxidative stress
Liu et al. (2008)	DMN-induced rat liver fibrosis	Complete YCHD decoction	Normal saline	4.18 g/kg/d (0.418 g/100g, oral)	↓ α-SMA, TGF-β1, TNF-α; ↓ CD68 (Kupffer cells); ↓ TIMP-1/2, MMP-2; ↑ MMP-9; reduced hydroxyproline; inhibited HSC activation
Cai et al. (2019)	DMN-induced rat liver fibrosis	Complete YCHD decoction	Normal saline	3.15 g/kg/d (oral)	↓ liver fibrosis; ↓ serum TNF-α; ↓ TUNEL-positive hepatocytes; ↓ cleaved caspase-3, Bax; ↑ Bcl-XL
Wu et al. (2019)	BDL rat	Complete YCHD decoction	Normal saline	10 g/kg/d (oral; 1 mL/100g, 1 g/mL)	↓ TBIL, DBIL, ALT, AST, GGT, ALP; inhibited PERK-CHOP-GADD34 pathway; restored Bax/Bcl-2 balance; reduced hepatocyte apoptosis

BDL, bile duct ligation; ANIT, α-naphthylisothiocyanate; DMN, dimethylnitrosamine; HSC, hepatic stellate cell.

Bile Duct Ligation (BDL) Model: Wu et al. (2019) divided 30 rats into control, OJ model (BDL), and YCHD treatment groups. The OJ model group exhibited unclear liver tissue texture, narrowed and congested hepatic sinusoids, disordered hepatic lobule architecture, as well as overt cholestasis, hepatocellular swelling, degeneration, and necrosis. Serum levels of total bilirubin (TBIL), direct bilirubin (DBIL), alanine aminotransferase (ALT), aspartate aminotransferase (AST), gamma-glutamyl transferase (GGT), and alkaline phosphatase (ALP) were significantly elevated. In contrast, YCHD treatment significantly alleviated these pathological changes and normalized the abnormal biochemical parameters. Mechanistically, YCHD suppressed the PERK-CHOP-GADD34 apoptosis pathway and restored the Bax/Bcl-2 balance (Wu et al., 2019). In another BDL study, Liu et al. (2025) demonstrated that YCHD mitigated liver injury by inhibiting epidermal growth factor receptor (EGFR) activation, promoting constitutive androstane receptor (CAR) nuclear translocation, and upregulating bile acid-metabolizing enzymes (CYP3A, UGT1A1, SULT2A1) and transporters (MRP2, MRP3, MRP4).

ANIT-Induced Cholestasis Model: Yi et al. (2018) showed that in ANIT-treated rats, YCHD restored the expression of UGT1A1, MRP2, BSEP, and OATP1A4, thereby promoting bilirubin and bile acid excretion. Li et al. (2025b) further employed a lipidomics and metabolomics approach in ANIT-induced rats and found that YCHD metabolites (e.g., emodin, geniposide, quercetin) regulated the PPARγ/NF-κB/JNK pathway, suppressed inflammation, and restored glycerolipid, glycerophospholipid, and amino acid metabolism. Additionally, Liu J-J. et al. (2022) demonstrated that YCHD activated the Nrf2 pathway in ANIT-treated rats, promoting nuclear translocation of Nrf2 and upregulating antioxidant enzymes such as NQO1 and GST, thereby limiting oxidative injury.

Gut-Liver Axis Studies: Li et al. (2025a) recently reported that in a mouse model of cholestasis, YCHD remodels the intestinal flora to enrich secondary bile acids (e.g., CDCA, DCA, CA) that activate the intestinal FXR-FGF15 axis, subsequently inhibiting hepatic Cyp7a1 expression and reducing bile acid synthesis. YCHD also preserved intestinal barrier integrity and limited bacterial and toxin translocation.

Fibrosis Models: In a dimethylnitrosamine (DMN)-induced liver fibrosis model, Liu et al. (2008) found that YCHD reduced hepatic stellate cell (HSC) activation (evidenced by decreased α-SMA expression) and modulated the inflammatory microenvironment by downregulating CD68 and profibrotic factors (TGF-β1, TNF-α).

#### 4.1.2 *In vitro* cell models

Complementing the *in vivo* findings, several *in vitro* studies have dissected the direct effects of YCHD or its active metabolites on specific cell types involved in OJ pathogenesis.

Hepatocytes: Cai et al. (2019) used TNF-α-treated hepatocytes to model apoptosis in cholestasis. They identified that YCHD metabolites such as emodin and quercetin suppressed TNF-α-induced apoptosis by modulating the PI3K-Akt pathway, reducing cleaved caspase-3, and increasing p-ERK1/2, PI3K, and Bcl-XL.

Hepatic Stellate Cells (HSCs) and Kupffer Cells: Primary HSCs or HSC cell lines have been used to study fibrogenesis. Liu et al. (2008) demonstrated that YCHD-containing serum inhibited HSC activation and reduced α-SMA and collagen I expression, consistent with their *in vivo* observations. Regarding Kupffer cells, although direct *in vitro* studies are limited, the downregulation of CD68 (a Kupffer cell marker) in YCHD-treated animals (Liu et al., 2008) suggests that YCHD may suppress Kupffer cell-mediated inflammation, which warrants further *in vitro* validation.

Collectively, these preclinical studies across multiple animal and cell models provide robust evidence that YCHD exerts multi-target therapeutic effects against OJ, including regulating bile acid homeostasis, alleviating inflammatory and oxidative damage, inhibiting hepatocyte apoptosis, and preventing hepatic fibrosis.

### 4.2 Clinical evidence

Several meta-analyses and randomized controlled trials (RCTs) have explored the therapeutic efficacy of YCHD for OJ and cholestatic conditions. Shi et al. (2021) conducted a meta-analysis of 19 animal studies (404 animals) and confirmed that YCHD significantly reduced cholestasis indices and improved liver function parameters. Chen et al. (Chen et al., 2015) performed a meta-analysis incorporating 15 RCTs involving 1,405 cholestasis patients, comparing YCHD with conventional drugs (mainly UDCA). The results indicated that YCHD significantly reduced serum levels of cholestasis markers (ALT, AST, TBIL, and DBIL), with no reported adverse outcomes. Furthermore, Luo et al. (2024) employed a combined approach of network meta-analysis and network pharmacology to explore the therapeutic effects of adjusted YCHD ingredient ratios. This study screened 20 eligible studies involving 1,591 patients. The results showed that different ratios exhibited distinct therapeutic effects for different treatment purposes. For example, TG-c (Yinchen:Zhizi:Dahuang = 10:5:2 to 10:5:3) combined with conventional therapy emerged as the most promising strategy for improving AST and TBIL levels. For reducing AST specifically, TG-e (5:2:2 to 5:3:3) plus conventional therapy was the most effective regimen. Notably, this study also proposed that Zhizi and Dahuang may exert bidirectional effects, potentially increasing the risk of cholestasis and liver injury under certain conditions; therefore, their proportions may need adjustment in clinical practice.

Recent clinical trials (listed in Table 5) continue to investigate YCHD as an adjunctive therapy for intrahepatic cholestasis of pregnancy, hypercoagulability of pregnancy, and biliary cholangitis, often in combination with UDCA. These studies aim to optimize dosing, safety, and efficacy in real-world settings. Although the efficacy of YCHD has been confirmed in numerous clinical practices and studies, the existing clinical evidence has limitations, including small sample sizes, heterogeneity, lack of blinding, and short follow-up durations. High-quality, large-scale, multicenter RCTs with rigorous design are urgently needed to further validate the long-term safety and comparative effectiveness of YCHD against standard pharmacological therapies.

**TABLE 5:** Relevant clinical trial information of YCHD in the past 5 years.

Main ID	Study subjects	Experimental group	Control group	Sample size	Study type	Sponsor	Ref.
ChiCTR2500110984	Intrahepatic cholestasis during pregnancy	UDCA+YCHD	/	177	Retrospective	Tongde hospital of Zhejiang province	Chinese Clinical Trial Registry (2025)
ChiCTR2300068755	Dampness-heat and blood stasis type biliary cholangitis	UDCA+Jiawei YCHD	UDCA+Placebo	66	Interventional study	Gongli hospital of Shanghai pudong new area	Chinese Clinical Trial Registry (2023)
ChiCTR2400088564	Gestational hypercholanemia	UDCA+Jiawei YCHD	UDCA	170	Interventional study	Department of gynecology and obstetrics hospital affiliated to Zhejiang university medical college	Chinese Clinical Trial Registry (2024)

## 5 Discussion

YCHD is a time-honored formula renowned both historically and contemporarily, demonstrating favorable therapeutic effects in treating cholestasis, chronic hepatitis, non-alcoholic fatty liver disease, and hepatic carcinoma (Xu et al., 2019; Zhang et al., 2020; Sun J. et al., 2021; Yang et al., 2025; Xu et al., 2026). Like many traditional TCM formulas, YCHD is characterized by multi-component and multi-target actions. While this is its unique advantage, it also limits the standardization of its clinical application to a certain extent (Xu et al., 2024; Li, 2020). This study summarizes and analyzes the current research on YCHD adjuvant therapy for OJ from three dimensions: component analysis, mechanistic investigation, and preclinical/clinical practice. We propose that this analytical framework also provides a paradigm for further understanding the therapeutic mechanisms of YCHD and other TCM formulas.

In terms of component analysis, research has evolved from *in vitro* component identification to animal serum detection, and further to the identification of enriched metabolites in target tissues/organs, gradually focusing on core active substances. Pharmacokinetic studies have identified nine candidate active metabolites absorbed into the bloodstream, including metabolites of scoparone and key compounds such as geniposide, rhein, and emodin (Wang et al., 2008). Notably, the complete YCHD formula consistently exhibits greater efficacy than any single component or combination of two metabolites, supporting the synergistic effects of the multi-herb formula (Wang et al., 2013). Future studies should move beyond qualitative detection to achieve quantitative assessment of component enrichment in target tissues under pathological conditions. At the mechanistic level, three core mechanisms of YCHD’s efficacy have been summarized: regulating bile acid metabolism (via EGFR-CAR and FXR-FGF15 axes, as well as BA transporters) (Yi et al., 2018), modulating inflammatory and metabolic responses (via PPARγ/NF-κB/JNK and Nrf2 pathways) (Li et al., 2025b), and protecting the liver while inhibiting fibrosis and apoptosis (via PERK-CHOP-GADD34, PI3K-Akt, and Bax/Bcl-2 pathways) (Cai et al., 2019). These mechanisms directly target the diverse pathological changes induced by OJ, fully demonstrating the therapeutic potential of YCHD.

In preclinical and clinical practice, animal experiments have confirmed the jaundice-reducing and hepatoprotective effects of YCHD, and meta-analyses have provided evidence-based support for its efficacy (Shi et al., 2021). Luo et al. (2024) pointed out that Zhizi and Dahuang exert bidirectional effects in YCHD, which may induce adverse effects under specific conditions, and found that adjusting the ratio to reduce the proportions of these two botanical drugs yields superior comprehensive therapeutic efficacy. However, the existing clinical evidence has notable limitations, including small sample sizes, heterogeneity, lack of blinding, and short follow-up durations. Systematic long-term pharmacovigilance data are lacking, and claims of “high safety and low toxicity” require further validation.

Several knowledge gaps remain to be addressed. Mechanistically, future research should move beyond qualitative or serum-based pharmacokinetics to achieve quantitative assessment of component enrichment in target tissues under pathological conditions. It is also essential to define the causal relationships between specific active substances and their purported mechanisms, and to empirically verify synergistic interactions among metabolites. Clinically, the priority is to conduct rigorous, adequately powered randomized controlled trials that incorporate direct comparisons between different YCHD ratios and standard care, with robust blinding and bias control. Concurrently, systematic studies on long-term safety and strategies to reduce potential toxicity (e.g., from specific metabolites like Zhizi and Dahuang in certain ratios) are urgently needed. Furthermore, YCHD holds significant value for further exploration and development in several aspects. In terms of research domains: accumulating evidence has established a close association between the intestinal flora and various human diseases, with unique roles in disease treatment (Liu and Yang, 2023; Che et al., 2022). The intestinal flora has shown great potential in mediating the regulatory effects of YCHD (Wang et al., 2024; Ren et al., 2025; Chen et al., 2026). Future research should focus more on the interactions between YCHD metabolites and the intestinal flora. The integration of nanotechnology with TCM offers advantages such as precise delivery and enhanced efficacy (Gu et al., 2024; Wang et al., 2025). Future research could explore combining nanotechnology with YCHD to further improve therapeutic outcomes. Additionally, modern technologies such as high content screening (HCS) (Wang et al., 2019) can be employed to streamline processes such as component screening, target validation, and toxicity prediction. Integrative Chinese and Western medicine therapy also holds tremendous potential, but standardized treatment protocols and comprehensive clinical trials for integrative therapy with YCHD remain lacking. We believe that through sustained efforts, standardized and efficient implementation of integrative medicine can be achieved, enabling TCM to make greater contributions to global health.

## References

[B1] Ahsan N. Shariq M. Surolia A. Raj R. Khan M. F. Kumar P. (2024). Multipronged regulation of autophagy and apoptosis: emerging role of TRIM proteins. Cell. Mol. Biol. Lett. 29, 13. 10.1186/s11658-023-00528-8 38225560 PMC10790450

[B2] Alabdul R. I. El Naamani H. Dimitrov D. Morin R. Jaber B. L. (2025). Bile cast nephropathy: a systematic review of case reports and case series. World J. Hepatol. U. S. 17, 105120. 10.4254/wjh.v17.i4.105120 PMC12038420 40308820

[B3] Alaei Faradonbeh F. Lastuvkova H. Cermanova J. Hroch M. Nova Z. Uher M. (2022). Multidrug resistance-associated protein 2 deficiency aggravates estrogen-induced impairment of bile acid metabolomics in rats. Front. Physiol. 13, 859294. 10.3389/fphys.2022.859294 35388287 PMC8979289

[B4] Aranda J. F. Madrigal-Matute J. Rotllan N. Fernández-Hernando C. (2013). MicroRNA modulation of lipid metabolism and oxidative stress in cardiometabolic diseases. Free Radic. Biol. Med. 64, 31–39. 10.1016/j.freeradbiomed.2013.07.014 23871755 PMC4145589

[B5] Bao Y. Osowiecka M. Ott C. Tziraki V. Meusburger L. Blaßnig C. (2025). Dietary oxidized lipids in redox biology: oxidized olive oil disrupts lipid metabolism and induces intestinal and hepatic inflammation in C57BL/6J mice. Redox Biol. Neth. 81, 103575. 10.1016/j.redox.2025.103575 PMC11927754 40043451

[B6] Bhat A. A. Thapa R. Afzal O. Agrawal N. Almalki W. H. Kazmi I. (2023). The pyroptotic role of Caspase-3/GSDME signalling pathway among various cancer: a review. Int. J. Biol. Macromol. 242, 124832. 10.1016/j.ijbiomac.2023.124832 37196719

[B7] Cai F.-F. Bian Y.-Q. Wu R. Sun Y. Chen X.-L. Yang M.-D. (2019). Yinchenhao decoction suppresses rat liver fibrosis involved in an apoptosis regulation mechanism based on network pharmacology and transcriptomic analysis. Biomed. Pharmacother. 114, 108863. 10.1016/j.biopha.2019.108863 30991286

[B8] Cai Y. Zheng Q. Sun R. Wu J. Li X. Liu R. (2020). Recent progress in the study of artemisiae scopariae herba (yin chen), a promising medicinal herb for liver diseases. Biomed. Pharmacother. 130, 110513. 10.1016/j.biopha.2020.110513 32702631

[B9] Chandrashekhara S. H. Gamanagatti S. Singh A. Bhatnagar S. (2016). Current status of percutaneous transhepatic biliary drainage in palliation of malignant obstructive jaundice: a review. Indian J. Palliat. Care. U. S. 22, 378–387. 10.4103/0973-1075.191746 27803558 PMC5072228

[B10] Che Q. Luo T. Shi J. He Y. Xu D.-L. (2022). Mechanisms by which traditional Chinese medicines influence the intestinal flora and intestinal barrier. Front. Cell. Infect. Microbiol. Switz. 12, 863779. 10.3389/fcimb.2022.863779 35573786 PMC9097517

[B11] Chen Z. Ma X. Zhao Y. Wang J. Zhang Y. Li J. (2015). Yinchenhao decoction in the treatment of cholestasis: a systematic review and meta-analysis. J. Ethnopharmacol. Irel. 168, 208–216. 10.1016/j.jep.2015.03.058 25849734

[B12] Chen L. Li M. Yang Z. Tao W. Wang P. Tian X. (2020). Gardenia jasminoides ellis: ethnopharmacology, phytochemistry, and pharmacological and industrial applications of an important traditional Chinese medicine. J. Ethnopharmacol. Irel. 257, 112829. 10.1016/j.jep.2020.112829 32311486

[B13] Chen D. Li X. Fu J. Ma Y. Jiang H. Li Q. (2026). Clinical efficacy of jiawei yinchenhao decoction combined with ursodeoxycholic acid and probiotics in the management of nonalcoholic fatty liver disease. Arab. J. Gastroenterol. Egypt S1687-1979 (26), 00007–00009. 10.1016/j.ajg.2026.01.007 41833459

[B14] Chinese Clinical Trial Registry (2023). Clinical study of jiawei yinchenhao decoction therapy on primary biliary cholangitis patients with dampness and heat stasis. Identifier ChiCTR2300068755. Regist. Available online at: https://www.chictr.org.cn/showproj.html?proj=189661 (Accessed April 27, 2026).

[B15] Chinese Clinical Trial Registry (2024). Study on the effects and mechanisms of jia wei yin chen hao tang in the treatment of hypercholanemia during pregnancy. Identifier ChiCTR2400088564. Regist. Available online at: https://www.chictr.org.cn/showproj.html?proj=222304 (Accessed April 27, 2026).

[B16] Chinese Clinical Trial Registry (2025). A retrospective comparative analysis of the therapeutic efficacy and perinatal outcomes in patients with intrahepatic cholestasis of pregnancy treated with a combination of traditional Chinese and Western medicine versus Western medicine alone. Identifier ChiCTR2500110984. Regist. Available online at: https://www.chictr.org.cn/showproj.html?proj=292377 (Accessed April 27, 2026).

[B17] Feldman A. G. Sokol R. J. (2019). Neonatal cholestasis: emerging molecular diagnostics and potential novel therapeutics. Nat. Rev. Gastroenterol. Hepatol. 16, 346–360. 10.1038/s41575-019-0132-z 30903105

[B18] Gao H. Jin Z. Bandyopadhyay G. Wang G. Zhang D. Rocha K. C. E. (2022). Aberrant iron distribution via hepatocyte-stellate cell axis drives liver lipogenesis and fibrosis. Cell. Metab. U. S. 34, 1201–1213.e5. 10.1016/j.cmet.2022.07.006 PMC9365100 35921818

[B19] Gao Q. Li G. Zu Y. Xu Y. Wang C. Xiang D. (2024). Ginsenoside Rg1 alleviates ANIT-Induced cholestatic liver injury by inhibiting hepatic inflammation and oxidative stress via SIRT1 activation. J. Ethnopharmacol. Irel. 319, 117089. 10.1016/j.jep.2023.117089 37634749

[B20] Gu W. Kong R. Qi S. Cheng X. Cai X. Zhou Z. (2024). Sono-assembly of ellagic acid into nanostructures significantly enhances aqueous solubility and bioavailability. Food Chem. Engl. 442, 138485. 10.1016/j.foodchem.2024.138485 38278106

[B21] Horn P. Tacke F. (2024). Metabolic reprogramming in liver fibrosis. Cell. Metab. U. S. 36, 1439–1455. 10.1016/j.cmet.2024.05.003 38823393

[B22] Hui Y. Wang X. Yu Z. Fan X. Cui B. Zhao T. (2020). Scoparone as a therapeutic drug in liver diseases: pharmacology, pharmacokinetics and molecular mechanisms of action. Pharmacol. Res. 160, 105170. 10.1016/j.phrs.2020.105170 32877694

[B23] Jiang M. Zhao S. Yang S. Lin X. He X. Wei X. (2020a). An “essential herbal medicine”-licorice: a review of phytochemicals and its effects in combination preparations. J. Ethnopharmacol. Irel. 249, 112439. 10.1016/j.jep.2019.112439 31811935

[B24] Jiang M. Qi L. Li L. Li Y. (2020b). The caspase-3/GSDME signal pathway as a switch between apoptosis and pyroptosis in cancer. Cell. Death Discov. U. S. 6, 112. 10.1038/s41420-020-00349-0 PMC7595122 33133646

[B25] Jorge G. D. L. Tártaro R. R. Escanhoela C. A. F. Boin I. D. (2016). Long-time choledochal clamping in wistar rats causes biliary obstruction progressing to hepatic fibrosis. Transpl. Proc. U. S. 48, 2375–2378. 10.1016/j.transproceed.2016.06.011 27742301

[B26] Kang L.-M. Xu L. Yu F.-K. Zhang F.-W. Lang L. (2024). Advances in minimally invasive treatment of malignant obstructive jaundice. World J. Gastrointest. Surg. 16, 3650–3654. 10.4240/wjgs.v16.i12.3650 39734452 PMC11650242

[B27] Keppler D. (2014). The roles of MRP2, MRP3, OATP1B1, and OATP1B3 in conjugated hyperbilirubinemia. Drug Metab. Dispos. 42, 561–565. 10.1124/dmd.113.055772 24459177

[B28] Liu J.-J. Mei H.-W. Jing Y.-Y. Li Z.-L. Wu S.-G. Yuan H.-X. (2025). Yinchenhao decoction alleviates obstructive jaundice liver injury by modulating epidermal growth factor receptor and constitutive androstane receptor signaling. World J. Hepatol. 17, 101724. 10.4254/wjh.v17.i3.101724 40177192 PMC11959654

[B29] Li H. (2020). Advances in anti hepatic fibrotic therapy with traditional Chinese medicine herbal formula. J. Ethnopharmacol. Irel. 251, 112442. 10.1016/j.jep.2019.112442 31891799

[B30] Li J.-Y. Cao H.-Y. Sun L. Sun R.-F. Wu C. Bian Y.-Q. (2017). Therapeutic mechanism of yīn-chén-hāo decoction in hepatic diseases. World J. Gastroenterol. U. S. 23, 1125–1138. 10.3748/wjg.v23.i7.1125 28275293 PMC5323438

[B31] Li G. Xu Y. Gao Q. Guo S. Zu Y. Wang X. (2022). Ginsenosides restore lipid and redox homeostasis in mice with intrahepatic cholestasis through SIRT1/AMPK pathways. Nutrients 14, 3938. 10.3390/nu14193938 36235592 PMC9571347

[B32] Li W. Huang D. Luo Z. Zhou T. Jin Z. (2025a). Yinchenhao decoction mitigates cholestatic liver injury in mice *via* gut microbiota regulation and activation of FXR-FGF15 pathway. Pharm. (Basel). Switz. 18, 932. 10.3390/ph18070932 40732223 PMC12300480

[B33] Li W. Zhu J. Zhou T. Jin Z. (2025b). Exploring the mechanisms of yinchenhao decoction against ANIT-Induced cholestatic liver injury by lipidomics, metabolomics and network pharmacology. J. Pharm. Biomed. Anal. Engl. 258, 116736. 10.1016/j.jpba.2025.116736 39914330

[B34] Liu S. Yang X. (2023). Intestinal flora plays a role in the progression of hepatitis-cirrhosis-liver cancer. Front. Cell. Infect. Microbiol. 13, 1140126. 10.3389/fcimb.2023.1140126 36968098 PMC10034054

[B35] Liu C. Sun M. Yan X. Han L. Zhang Y. Liu C. (2008). Inhibition of hepatic stellate cell activation following yinchenhao decoction administration to dimethylnitrosamine-treated rats. Hepatol. Res. Neth. 38, 919–929. 10.1111/j.1872-034X.2008.00346.x 18371158

[B36] Liu W. Jing Z.-T. Xue C.-R. Wu S.-X. Chen W.-N. Lin X.-J. (2019). PI3K/AKT inhibitors aggravate death receptor-mediated hepatocyte apoptosis and liver injury. Toxicol. Appl. Pharmacol. U. S. 381, 114729. 10.1016/j.taap.2019.114729 31445927

[B37] Liu J. Qu J. Chen H. Ge P. Jiang Y. Xu C. (2021). The pathogenesis of renal injury in obstructive jaundice: a review of underlying mechanisms, inducible agents and therapeutic strategies. Pharmacol. Res. 163, 105311. 10.1016/j.phrs.2020.105311 33246170

[B38] Liu S. Yin R. Yang Z. Wei F. Hu J. (2022a). The effects of rhein on D-GalN/LPS-induced acute liver injury in mice: results from gut microbiome-metabolomics and host transcriptome analysis. Front. Immunol. Switz. 13, 971409. 10.3389/fimmu.2022.971409 PMC9648667 36389730

[B39] Liu J.-J. Xu Y. Chen S. Hao C.-F. Liang J. Li Z.-L. (2022b). The mechanism of yinchenhao decoction in treating obstructive-jaundice-induced liver injury based on Nrf2 signaling pathway. World J. Gastroenterol. 28, 4635–4648. 10.3748/wjg.v28.i32.4635 36157920 PMC9476870

[B40] Liu J.-J. Sun Y.-M. Xu Y. Mei H.-W. Guo W. Li Z.-L. (2023). Pathophysiological consequences and treatment strategy of obstructive jaundice. World J. Gastrointest. Surg. 15, 1262–1276. 10.4240/wjgs.v15.i7.1262 37555128 PMC10405123

[B41] Lu J.-Z. Ye D. Ma B.-L. (2021). Constituents, pharmacokinetics, and pharmacology of Gegen-Qinlian decoction. Front. Pharmacol. Switz. 12, 668418. 10.3389/fphar.2021.668418 PMC8139575 34025427

[B42] Luo S. Huang M. Lu X. Zhang M. Xiong H. Tan X. (2024). Optimized therapeutic potential of yinchenhao decoction for cholestatic hepatitis by combined network meta-analysis and network pharmacology. Phytomedicine 129, 155573. 10.1016/j.phymed.2024.155573 38583348

[B43] Ma S. Wei T. Zhang B. Zhang Y. Lai J. Qu J. (2024). Integrated pharmacokinetic properties and tissue distribution of multiple active constituents in Qing-Yi recipe: a comparison between granules and decoction. Phytomedicine. Ger. 129, 155645. 10.1016/j.phymed.2024.155645 38643714

[B44] Molinaro A. Marschall H.-U. (2022). Bile acid metabolism and FXR-Mediated effects in human cholestatic liver disorders. Biochem. Soc. Trans. Engl. 50, 361–373. 10.1042/BST20210658 35191955

[B45] Muchova L. Vanova K. Suk J. Micuda S. Dolezelova E. Fuksa L. (2015). Protective effect of heme oxygenase induction in ethinylestradiol-induced cholestasis. J. Cell. Mol. Med. 19, 924–933. 10.1111/jcmm.12401 25683492 PMC4420596

[B46] Pavlidis E. T. Pavlidis T. E. (2018). Pathophysiological consequences of obstructive jaundice and perioperative management. Hepatobiliary Pancreat. Dis. Int. 17, 17–21. 10.1016/j.hbpd.2018.01.008 29428098

[B47] Perez M.-J. Briz O. (2009). Bile-acid-induced cell injury and protection. World J. Gastroenterol. 15, 1677–1689. 10.3748/wjg.15.1677 19360911 PMC2668773

[B48] Radun R. Trauner M. (2021). Role of FXR in bile acid and metabolic homeostasis in NASH: pathogenetic concepts and therapeutic opportunities. Semin. Liver Dis. U. S. 41, 461–475. 10.1055/s-0041-1731707 34289507 PMC8492195

[B49] Ramírez-Pérez O. Cruz-Ramón V. Chinchilla-López P. Méndez-Sánchez N. (2017). The role of the gut microbiota in bile acid metabolism. Ann. Hepatol. Mex. 16, s15–s20. 10.5604/01.3001.0010.5494 29080339

[B50] Rana R. Shearer A. M. Fletcher E. K. Nguyen N. Guha S. Cox D. H. (2019). PAR2 controls cholesterol homeostasis and lipid metabolism in nonalcoholic fatty liver disease. Mol. Metab. 29, 99–113. 10.1016/j.molmet.2019.08.019 31668396 PMC6742970

[B51] Ren S. Gao Y. Wang J. Feng J. Li J. Xiang T. (2025). Yinchenhao decoction and its active compound rhein ameliorate intrahepatic cholestasis of pregnancy in mice via modulation of intestinal flora. Phytomedicine. Ger. 148, 157397. 10.1016/j.phymed.2025.157397 41135275

[B52] Rizzo A. Ricci A. D. Frega G. Palloni A. De Lorenzo S. Abbati F. (2020). How to choose between percutaneous transhepatic and endoscopic biliary drainage in malignant obstructive jaundice: an updated systematic review and meta-analysis. Vivo. Greece 34, 1701–1714. 10.21873/invivo.11964 32606139 PMC7439916

[B53] Russell D. W. (2003). The enzymes, regulation, and genetics of bile acid synthesis. Annu. Rev. Biochem. 72, 137–174. 10.1146/annurev.biochem.72.121801.161712 12543708

[B54] Shi P. Lin X. Yao H. (2018). A comprehensive review of recent studies on pharmacokinetics of traditional Chinese medicines (2014-2017) and perspectives. Drug Metab. Rev. Engl. 50, 161–192. 10.1080/03602532.2017.1417424 29258334

[B55] Shi K. Wen J. Zeng J. Guo Y. Hu J. Li C. (2021). Preclinical evidence of yinchenhao decoction on cholestasis: a systematic review and meta-analysis of animal studies. Phytother. Res. 35, 138–154. 10.1002/ptr.6806 32975338

[B56] Soltani A. Shams Abadi M. S. Raeisi M. Kouhihabibidehkordi G. Eshaghi F. Mohreh O. (2023). Apoptosis-inducing plant-based phenolic compounds are effective on leukemia cell lines. Curr. Pharm. Des. United Arab. Emir. 29, 1092–1104. 10.2174/1381612829666230417110032 37070446

[B57] Sun L. Cai J. Gonzalez F. J. (2021a). The role of farnesoid X receptor in metabolic diseases, and gastrointestinal and liver cancer. Nat. Rev. Gastroenterol. Hepatol. 18, 335–347. 10.1038/s41575-020-00404-2 33568795

[B58] Sun J. Han T. Yang T. Chen Y. Huang J. (2021b). Interpreting the molecular mechanisms of yinchenhao decoction on hepatocellular carcinoma through absorbed metabolites based on network pharmacology. Biomed. Res. Int. U. S. 2021, 6616908. 10.1155/2021/6616908 PMC8159653 34104649

[B59] Suraweera D. Rahal H. Jimenez M. Viramontes M. Choi G. Saab S. (2017). Treatment of primary biliary cholangitis ursodeoxycholic acid non-responders: a systematic review. Liver Int. U. S. 37, 1877–1886. 10.1111/liv.13477 28517369

[B60] Susukida T. Sekine S. Ogimura E. Aoki S. Oizumi K. Horie T. (2015). Basal efflux of bile acids contributes to drug-induced bile acid-dependent hepatocyte toxicity in rat sandwich-cultured hepatocytes. Toxicol Vitro 29, 1454–1463. 10.1016/j.tiv.2015.06.004 26055650

[B61] Wang X. Sun W. Sun H. Lv H. Wu Z. Wang P. (2008). Analysis of the constituents in the rat plasma after oral administration of yin chen hao tang by UPLC/Q-TOF-MS/MS. J. Pharm. Biomed. Anal. Engl. 46, 477–490. 10.1016/j.jpba.2007.11.014 18164893

[B62] Wang X. Sun H. Zhang A. Jiao G. Sun W. Yuan Y. (2011). Pharmacokinetics screening for multi-metabolites absorbed in the rat plasma after oral administration traditional Chinese medicine formula yin-chen-hao-tang by ultra performance liquid chromatography-electrospray ionization/quadrupole-time-of-flight mass spectrometry combined with pattern recognition methods. Anal. Engl. 136, 5068–5076. 10.1039/c1an15752c 21991580

[B63] Wang X. Zhang A. Wang P. Sun H. Wu G. Sun W. (2013). Metabolomics coupled with proteomics advancing drug discovery toward more agile development of targeted combination therapies. Mol. Cell. Proteomics 12, 1226–1238. 10.1074/mcp.M112.021683 23362329 PMC3650334

[B64] Wang J. Wu M.-Y. Tan J.-Q. Li M. Lu J.-H. (2019). High content screening for drug discovery from traditional Chinese medicine. Chin. Med. 14, 5. 10.1186/s13020-019-0228-y 30858873 PMC6394041

[B65] Wang Y. Chen J. Tang J. Xiao J. Zheng Y. Tang L. (2021). Combined LC-MS/MS and 16S rDNA analysis on mice under high temperature and humidity and herb yinchen protection mechanism. Sci. Rep. 11, 5099. 10.1038/s41598-021-84694-9 33658635 PMC7930127

[B66] Wang J. Ouyang B. Cao R. Xu Y. (2024). An UHPLC-QTOF-MS-based strategy for systematic profiling of chemical constituents and associated in vivo metabolites of a famous traditional Chinese medicine formula, yinchenhao decoction. Biomed. Chromatogr. 38, e5784. 10.1002/bmc.5784 38009806

[B67] Wang C. Ren K. Yang M. Li X. Li N. Li P. (2025). How traditional Chinese medicine can play a role in nanomedicine? A comprehensive review of the literature. Int. J. Nanomedicine. N. Z. 20, 6289–6315. 10.2147/IJN.S518610 40416728 PMC12103218

[B68] Wu L. Zhou P.-Q. Xie J.-W. Zhu R. Zhou S.-C. Wang G. (2015). Effects of yinchenhao decoction on self-regulation of renin-angiotensin system by targeting angiotensin converting enzyme 2 in bile duct-ligated rat liver. J. Huazhong Univ. Sci. Technol. Med. Sci. China 35, 519–524. 10.1007/s11596-015-1463-9 26223920

[B69] Wu Y.-L. Li Z.-L. Zhang X.-B. Liu H. (2019). Yinchenhao decoction attenuates obstructive jaundice-induced liver injury and hepatocyte apoptosis by suppressing protein kinase RNA-Like endoplasmic reticulum kinase-induced pathway. World J. Gastroenterol. U. S. 25, 6205–6221. 10.3748/wjg.v25.i41.6205 31749592 PMC6848016

[B70] Wu H. Zhang Y. Liang J. Wu J. Zhang Y. Su H. (2023). Lithium chloride induces apoptosis by activating endoplasmic reticulum stress in pancreatic cancer. Transl. Oncol. 38, 101792. 10.1016/j.tranon.2023.101792 37806114 PMC10579530

[B71] Xiang D. Yang J. Liu L. Yu H. Gong X. Liu D. (2023). The regulation of tissue-specific farnesoid X receptor on genes and diseases involved in bile acid homeostasis. Biomed. Pharmacother. Fr. 168, 115606. 10.1016/j.biopha.2023.115606 37812893

[B72] Xu L. Xie T. Shen T. Jian S. (2019). Yinchenhao decoction for chronic hepatitis B: protocol for a systematic review and meta-analysis. Med. Baltim. 98, e14648. 10.1097/MD.0000000000014648 30813205 PMC6408079

[B73] Xu F. Zhang H. Chen J. Zhan J. Liu P. Liu W. (2024). Recent progress on the application of compound formulas of traditional Chinese medicine in clinical trials and basic research in vivo for chronic liver disease. J. Ethnopharmacol. Irel. 321, 117514. 10.1016/j.jep.2023.117514 38042388

[B74] Xu F.-P. Yang C. Yu S.-H. Cai H. Hui M.-Y. Fang L.-L. (2026). Yinchenhao decoction, a compound Chinese herbal medicine, for patients with nonalcoholic fatty liver disease: a randomized, double-blind, placebo-controlled trial. J. Integr. Med. S2095-4964 (26), 00035–X. 10.1016/j.joim.2026.03.003 41905832

[B75] Yang R. Jiang D. Xu H. Yang H. Feng L. Wu Q. (2025). Network pharmacology and molecular docking integrated with molecular dynamics simulations investigate the pharmacological mechanism of yinchenhao decoction in the treatment of non-alcoholic fatty liver disease. Curr. Comput. Aided Drug Des. United Arab. Emir. 21, 721–737. 10.2174/0115734099305489240702075128 38994616

[B76] Yi Y.-X. Ding Y. Zhang Y. Ma N.-H. Shi F. Kang P. (2018). Yinchenhao decoction ameliorates alpha-naphthylisothiocyanate induced intrahepatic cholestasis in rats by regulating phase II metabolic enzymes and transporters. Front. Pharmacol. Switz. 9, 510. 10.3389/fphar.2018.00510 29867509 PMC5962729

[B77] Zhang J. Liu X. Wu J. Zhou W. Tian J. Guo S. (2020). A bioinformatics investigation into the pharmacological mechanisms of the effect of the yinchenhao decoction on hepatitis C based on network pharmacology. BMC Complement. Med. Ther. Engl. 20, 50. 10.1186/s12906-020-2823-y 32050950 PMC7076901

[B78] Zhang P. Jiang N. Xu L. Shen Z. Liu X. Cai X. (2022). *Clostridium perfringens* and *Escherichia coli* bacteremia in a patient with acute obstructive suppurative cholangitis: a case report and review of the literature. Am. J. Case Rep. 23, e936329. 10.12659/AJCR.936329 35526110 PMC9096897

[B79] Zhuang T. Gu X. Zhou N. Ding L. Yang L. Zhou M. (2020). Hepatoprotection and hepatotoxicity of Chinese herb rhubarb (dahuang): how to properly control the “General (Jiang Jun)” in Chinese medical herb. Biomed. Pharmacother. 127, 110224. 10.1016/j.biopha.2020.110224 32559851

